# Promoting equity, inclusion, and efficiency: A team science approach to the development of authorship guidelines for a multi-disciplinary research team

**DOI:** 10.1017/cts.2023.685

**Published:** 2023-11-30

**Authors:** Hannah Lewis, Barbara Biesecker, Sandra Soo-Jin Lee, Katherine Anderson, Galen Joseph, Charisma L. Jenkins, Joanna E. Bulkley, Michael C. Leo, Katrina A. B. Goddard, Benjamin S. Wilfond

**Affiliations:** 1 Treuman Katz Center for Pediatric Bioethics, Seattle Children’s Research Institute, Seattle, WA, USA; 2 RTI International, Research Triangle Park, NC, USA; 3 Department of Medical Humanities and Ethics, Columbia University, New York, NY, USA; 4 Denver Health Medical Center, Denver, CO, USA; 5 Department of Humanities and Social Sciences, University of California, San Francisco, CA, USA; 6 Department of Translational and Applied Genomics, Kaiser Permanente Center for Health Research, Portland, OR, USA; 7 Division of Cancer Control and Population Sciences, National Cancer Institute, National Institutes of Health, Rockville, MD, USA; 8 Department of Pediatrics, Division of Bioethics and Palliative Care, University of Washington, Seattle, WA, USA

**Keywords:** Team science, authorship, multidisciplinary research, equity

## Abstract

Large research teams and consortia present challenges for authorship. The number of disciplines involved in the research can further complicate approaches to manuscript development and leadership. The CHARM team, representing a multi-disciplinary, multi-institutional genomics implementation study, participated in facilitated discussions inspired by team science methodologies. The discussions were centered on team members’ past experiences with authorship and perspectives on authorship in a large research team context. Team members identified challenges and opportunities that were used to create guidelines and administrative tools to support manuscript development. The guidelines were organized by the three values of equity, inclusion, and efficiency and included eight principles. A visual dashboard was created to allow all team members to see who was leading or involved in each paper. Additional tools to promote equity, inclusion, and efficiency included providing standardized project management for each manuscript and making “concept sheets” for each manuscript accessible to all team members. The process used in CHARM can be used by other large research teams and consortia to equitably distribute lead authorship opportunities, foster coauthor inclusion, and efficiently work with large authorship groups.

## Introduction

Advances in clinical research are increasingly generated by large multi-disciplinary teams, and sometimes these teams are organized into even larger groups such as research consortia. Distributing authorship opportunities amongst the members of these large groups and identifying those who prefer or even expect to be included in writing manuscripts can pose a challenge. Factors that contribute to these challenges include differences in disciplinary norms, institutional culture, and career status [1–4]. Less experienced team members may be unfamiliar with the range of publication practices and may find it difficult to navigate authorship roles [4,5]. Nevertheless, there has been growing appreciation of the importance of equity and inclusion in manuscript development to promote the professional growth and work satisfaction of team members [6,7].

The International Committee of Medical Journal Editors (ICMJE) has developed recommendations that provide criteria regarding what are sufficient contributions for authorship [8]. These guidelines provide an important framework for confirming authorship criteria have been met at the time of submission. However, they do not offer prospective guidance for who should lead the manuscript and who should be involved in its development. In addition, while previous literature addresses authorship disputes within multi-disciplinary research teams and what is considered sufficient criterion for authorship [9–13], less has been written about how large research teams can engage in these prospective processes that provide structure and clarity to authorship roles and responsibilities, thereby sharing authorship opportunities equitably. Furthermore, manuscript preparation can become progressively time-intensive and burdensome to navigate as the size of the authorship group increases [14].

Team science is an organizational approach that aims to facilitate engagement within interdisciplinary teams to support team collaboration and mitigate conflict through communication and the development of standard processes [15]. In this manuscript, we describe how we used a team science approach to develop an equitable and inclusive authorship process for our large multi-disciplinary, multi-site research project.

## Materials and Methods

The CHARM study was a genomic medicine implementation study that sought to increase access to genomic testing for hereditary cancer syndromes for individuals from low-income, low-literacy, and Spanish-speaking populations [16]. The CHARM team consisted of approximately 75 multi-disciplinary members across ten institutions and several time zones. The team was organized into working groups with overlapping members, dedicated to different aspects of the study. Each working group had a designated “team lead” who was responsible for leading that aspect of the study, including major manuscript decisions, in collaboration with the principal investigators. CHARM is one of seven CSER (Clinical Sequencing Evidence-Generating Research) Consortium Studies organized by NHGRI [17]. Throughout the project period, CHARM held bi-weekly virtual meetings that all investigators and staff were encouraged to attend and actively participate in. Halfway through the study, discussions about manuscript planning during these meetings revealed variations in the expectations of team members and in team members’ previous experiences with authorship and manuscript development.

Recognizing the concerns team members expressed as manuscript development intensified, the CHARM Team Science Working Group was tasked with developing CHARM authorship guidelines. In the summer of 2020, the working group led four simultaneous virtual small group hour-long discussions on authorship with the team. Each session was moderated by a senior investigator (BB, GJ, SL, KA) with experience leading focus groups. A scribe was assigned to each group to take notes. Team members were purposively assigned to groups of 8–10 team members that included investigators and research staff across all institutions and working groups of the CHARM study. The moderators facilitated the discussion using four prompts: (1) “What does authorship mean to you? Why is it important?,” (2) “What has been your experience with authorship in the past (other studies and CHARM)?,” (3) “What challenges have you experienced?,” and (4) “What processes may help to address challenges that may arise?”

In the weeks following the small group sessions, the CHARM team convened for a large group discussion facilitated by BW to promote interactive discussion and elicit further feedback. The notes from the small and large group discussions were summarized and compiled by a project manager (HL). The challenges and suggestions identified were inductively organized into values and principles by our Team Science Working Group (BW, KG, HL, BB, SL, GJ) to guide the development of a standardized approach to authorship.

CHARM principal investigators (BW, KG) drafted an authorship guidelines document to address the specific authorship goals of the CHARM team. These guidelines drew from the equitable and inclusive authorship approach recently developed at the Kaiser Permanente Center for Health Research (unpublished internal institutional policy). A draft of the guidelines was presented to the CHARM team via email and at team meetings for feedback. Once the revised guidelines were circulated to the team, CHARM team members (TK, CJ, JB) developed specific processes and approaches to support the guidelines.

## Results

### CHARM Team Authorship Discussions

*The meaning of authorship:* Most team members viewed authorship as valuable to their career advancement and meeting performance goals. Authorship was also seen as especially valuable for those early in their career to gain experience and recognition, including the importance of taking on first and senior authorship roles during manuscript development. Some team members viewed authorship as an explicit expectation of their institutional role. Others, including some of those who were involved in specific tasks such as recruitment or genetic laboratory analysis, acknowledged the value of inclusion in some manuscripts to reflect their contributions, even if not an institutional expectation of their role. The discussion groups recognized that authorship expectations can differ among individuals, depending on one’s discipline, career stage, role within the study team, and/or institution. The expectations may be related to the meaning of authorship order, speed of publication, and co-authorship responsibilities.

*Past experiences and challenges with authorship*. One key challenge team members identified was determining who should be the lead authors, i.e. first and last (senior) authors. Team members reported past experiences that were, at times, both awkward and difficult, particularly when more than one team member perceived that they had contributed equally to the work related to the topic of the manuscript. Additionally, some team members noted that the traditional model of the project principal investigator being the senior author was problematic for this large multi-disciplinary study that involves diverse, multiple senior leadership. They also expressed some confusion as to who was responsible for determining the first and senior authors and their responsibilities for a manuscript.

The second key challenge involved inclusion as a coauthor. There was concern that in a large research team, many team members may meet authorship criteria, but it was challenging to identify these potential authors because of the fragmented way our work was conducted across multiple institutions and working groups. People working on the same project might never have any direct interaction with each other or be fully aware of who is engaged in moving the work forward. It was also unclear whether team members involved in core tasks, such as recruitment and data analysis, should always be included as coauthors, especially because it could be infeasible or burdensome for those team members given the number of manuscripts expected to be produced by the study. Some team members expressed frustration that there were occasions in which they were unaware of a manuscript that was in progress to which they would have liked to contribute. Some team members felt that to be included on a paper required an assertiveness that, on bigger teams, can feel uncomfortable or perceived as self-promotion. Conversely, team members also discussed the burden of having too many coauthors on a manuscript, which could substantially delay receiving feedback and lead to a greater likelihood of having to resolve contradictory feedback.

*Processes to address the challenges of authorship.* The small group participants recommended clarifying expectations for each authorship role in manuscript writing. Team members recommended using an acknowledgments section, or when allowed by a journal, a “non-author contributor” list, to recognize team members who made contributions to the research and did not have the capacity to be involved in fulfilling all responsibilities of co-authorship. CHARM team members further suggested creating a visual dashboard to display every team member’s involvement in each manuscript. This created an opportunity to improve equity of lead authorship and inclusion in authorship amongst team members.

Additional recommendations addressed the culture of authorship. Team members recommended that potential authors be transparent and communicate about their capacity to contribute to a manuscript. Team members suggested supporting one another to advocate for themselves in the writing process, while also recognizing that some are uncomfortable with advocating for themselves in a large research team. Team members also discussed the need for a process to moderate differences in approaches to a manuscript, calling for collaborative engagement between lead authors and team leads to deliberate options and arrive at a solution.

### Authorship Guidelines

Following the group discussions, recommendations were incorporated into existing tools and approaches and were also used to aid the development of new tools and approaches. For instance, at the outset of CHARM, the team created and implemented a *concept sheet* for each manuscript as a tool to define a manuscript’s scope, analysis plan, and audience as well as which team members may be involved (see supplementary material 1). The CHARM team leads were typically responsible for generating a concept sheet. The *Team leads* were the group of investigators who directed key aspects of the CHARM study, often representing different institutions, and including roles such as the multiple principal investigators, site principal investigators, program director (administrative lead), and co-investigators leading critical functions of the study. They had expertise in a variety of areas of practice (i.e., genetic counseling, laboratory science, patient engagement, health systems) and worked collaboratively with the study principal investigators and study methodologists (quantitative and/or qualitative) to create the CHARM analysis and dissemination plan, including determining the lead authors for each manuscript. The concept sheet process was incorporated by the Team Science Working Group into the authorship guidelines.

The guidelines included three components to support the authorship objectives of the CHARM team. One component clarified the roles and responsibilities of authors. A second described the CHARM study’s values and related principles to uphold these values. Further, the guidelines established several implementation approaches to support manuscript authorship decisions, writing processes, and management of the manuscript. The goal of the guidelines was to set expectations that promote a cohesive authorship culture within the CHARM team. They were intended to be inclusive in offering co-authorship to all who contribute to the research and to support junior researchers taking on leadership roles.

The guidelines defined author roles for each manuscript (see Table 1). The *lead authors* for each manuscript guided the analysis and writing and were typically the first and last authors. The *primary writing group* is the core group involved in the details of the manuscript development. The secondary writing group is not involved in drafting but meets authorship criteria under ICMJE guidelines due to their involvement in editing the manuscript.

**Table 1.** Authorship roles and responsibilities

Responsibilities	Lead authors	Primary writing group	Secondary writing group
Contribute to CHARM project in a way related to the manuscript	X	X	X
Draft concept sheet and share with CHARM team	X		
Identify journal and audience	X		
Organize writing team	X		
Attend meetings about manuscript	X	X	
Draft manuscript	X		
Might contribute to drafting manuscript		X	
Provide guidance to primary writing group			X
Review, edit/comment on drafts		X	X
Make editorial decisions	X		
Approve final version	X	X	X
Submit manuscript to journal	X		
Respond to reviewer comments	X	X	X

The responsibilities for authorship are based on each author’s role in the manuscript.

The CHARM guidelines articulate three core values of equity, inclusion, and efficiency with associated principles for each in authorship opportunities. In this context, equity refers to the distribution of leadership roles in manuscripts. Inclusion refers to including those team members who would like the opportunity to contribute to manuscripts and have made a sufficient contribution. Efficiency refers to developing processes to prospectively facilitate the development of the manuscript to reduce the overall time and effort required to produce the manuscript. Each value (equity, inclusion, and efficiency) is connected to principles and implementation approaches.

*Equity:* Our team identified three authorship principles related to equity. First, team leads should distribute lead authorship opportunities among team members who are guiding key aspects of the study. Second, team leads should encourage junior team members to be first author with mentorship from a senior author. Third, lead authors are also expected to identify second and third authors and up to two additional senior authors. The rationale for identifying these roles close to the onset of working on each manuscript was to clarify roles and broaden leadership within the team.

**Figure 1.**
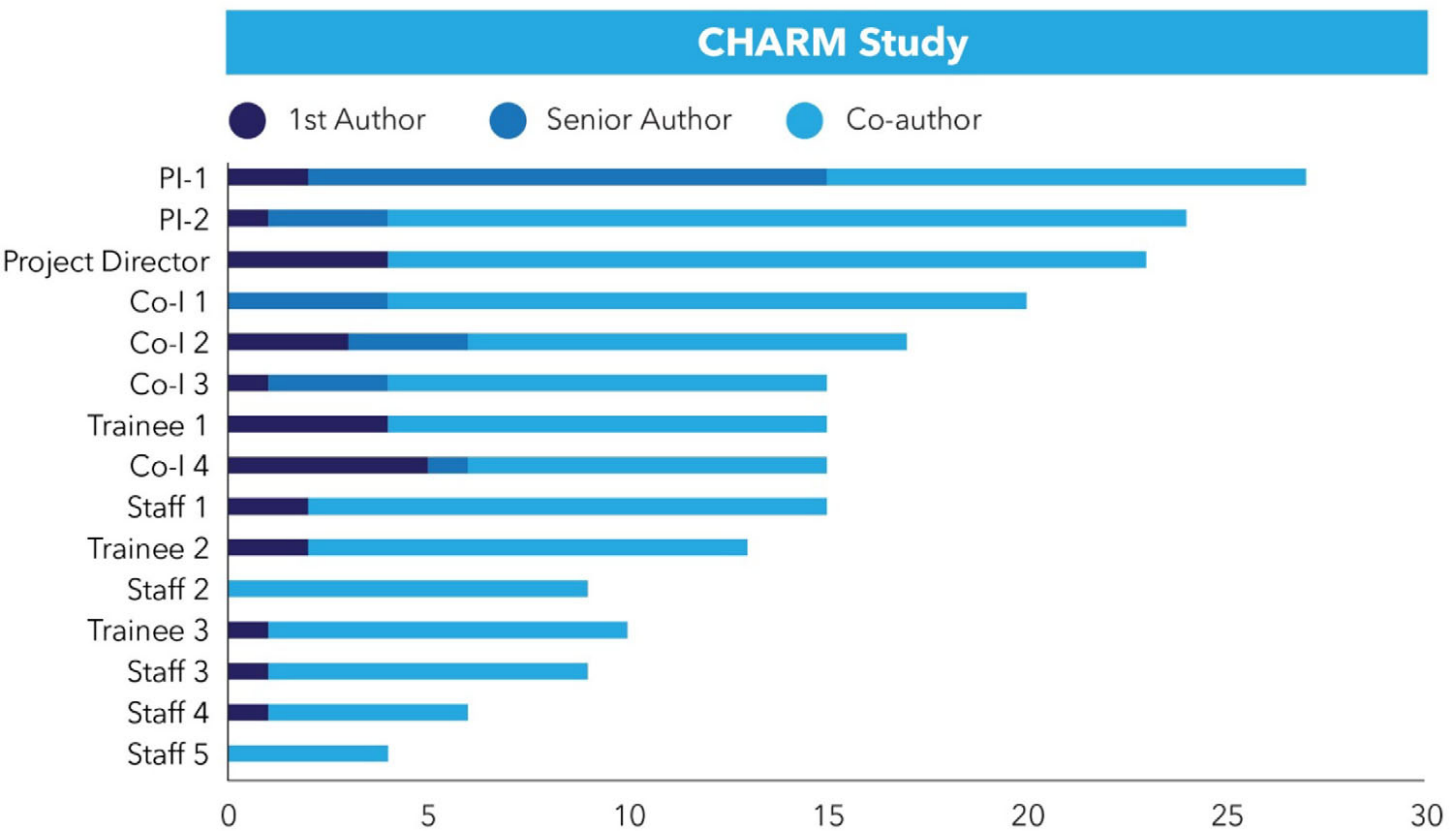
CHARM manuscript authorship distribution dashboard using Smartsheet. Illustration of how a visual dashboard can show individual team members and their role as a lead author (first author, last author) or coauthor. This illustration conveys that first authors' roles are distributed across investigators, trainees, and staff. The illustration also shows distribution of senior author roles across team members. Finally, the illustration shows that team members are included as coauthors with variable frequency.

We developed a tool in Smartsheet (CJ, JB), a cloud-based work management platform, which allowed the CHARM team to visualize who was involved in each of 34 CHARM manuscripts (ranging from planned to published) and authorship roles for each team member. The first author entered information into a form that populated the Smartsheet for each manuscript, including the authors, their role in the manuscript, and institutions. Monthly automated requests were sent to first authors asking that they update the information, which helps to capture changes to authorship lists during manuscript development. Data were displayed as a visual dashboard to summarize authorship distribution across the CHARM team. The dashboard listed all team members across the various CHARM sites and displayed a stacked bar chart showing the manuscripts and the authorship roles that have been filled by team members (see Fig. 1). The dashboard was accessible to all team members and promoted equity by providing a visual aid to identify which team members have or have not been lead authors. In addition, the dashboard promoted inclusion by identifying team members who might have been overlooked as a coauthor on each manuscript and promoted efficiency by tracking the progress of manuscripts and helping with the management of manuscripts across the project via automated update requests. The template the CHARM team created is available. (https://app.smartsheet.com/b/launch?lx=IG61eWKGzJYH-i8MNlVaiuqKwon7W423t4KaXJloEug). Our team created an overview of the approach, startup guide, and a data dictionary which are available as supplementary material 2.

*Inclusion*: The first principle is for team leads and lead authors to consider who has been involved in the research activities related to a manuscript when nominating team members to be coauthors. A second principle is for team leads and lead authors to ensure the larger team is aware of all manuscripts in progress.

Team leads and lead authors are expected to identify the writing teams on the concept sheet. The concept sheet is reviewed by the principal investigators and by all the team leads and is presented at team meetings. At each step, team members can nominate others or themselves for the paper. The concept sheets, as well as the dashboard, were accessible to all team members in cloud-based files to promote transparency in manuscript development. We also created a “non-author” contributor list of current and past team members that could be used on any paper, to be acknowledged “on behalf of the CHARM team.” This allowed those who have received funding support from the grant or have contributed significantly to the project to be recognized even if they were not authors on a paper.

*Efficiency:* The first efficiency principle advises team leads to be realistic about the capacity of the team members to lead papers. This is important to ensure that progress on a paper is made in a timely way. In addition, team leads are advised to focus on completing manuscripts that are of highest priority. We appreciated that this prioritization could allow us to write the most important manuscripts with more engagement of the team compared to what might happen if too many papers were planned to be written at the same time. A correlated second principle is for team members to be realistic about their own capacity to fulfill the obligations of being a lead author or part of a writing group when deciding whether to commit to work on a manuscript. Third, the coauthors should take responsibility to be actively engaged in the development and/or review of manuscripts, including communicating with the lead authors in a timely manner and proactively asking for more time, if needed.

To implement our efficiency principles, project managers were assigned to each manuscript to facilitate efficient development and review of manuscripts. These project managers established clear timelines and deadlines. They were expected to forecast expectations for each step to promote responsiveness to timelines by all authors. Lead authors were encouraged to limit the number of rounds of review. If a secondary author did not respond to deadlines, team leads were expected to follow up and keep the paper moving forward.

## Discussion

The CHARM team developed authorship guidelines by participating in a collaborative process [15]. The guidelines promote equity in lead/senior authorship opportunities, inclusion of team members as coauthors, and efficiency in manuscript writing. While the ICMJE [8] guidelines aid in the retrospective determination of authorship, the CHARM guidelines illustrate how study guidelines can be used to prospectively direct authorship roles to meet the ICMJE guidelines.

In addition to efficiency, the values of equity and inclusion have become increasingly recognized as essential to the success of academic pursuits. While the use of the concepts in our authorship guidelines differs from the more traditional meaning of advancing equity and inclusion by centering the importance of underrepresented team members in the research enterprise [18,19], using equity and inclusion as a lens for the development of these guidelines promotes leadership in manuscript development and promotes recognition of team member contributions. Our team chose to emphasize the equitable distribution of lead author responsibilities because of the value we place on personal and professional development of team members. We emphasized inclusion because of the value of acknowledging the contributions of our team members, the vast array of disciplines they represent, and the variety of expertise they contribute to the success of the research endeavor. However, our fundamental rationale is a belief that a manuscript development process that embraces equity and inclusion will result in better science and better training for the next generation of researchers. An approach to authorship that optimizes these values can still support an efficient manuscript process by expecting all coauthors to respond to requests for input in a timely manner, as well as additional strategies, including limiting the number of revisions, using a software platform that allows simultaneous revisions, or proposing a deliberate method for sequential revisions by each author.

Large research teams and consortia, such as the CHARM study, are uniquely positioned to produce multiple manuscripts to communicate the research activities. Investigation into multifaceted research questions can result in multiple papers that allow for broader opportunities for team members to lead or be included as a coauthor on a manuscript. Further, by using a non-author contributor list, additional team members can be recognized for their contribution to the success of the research project, although they may have not been able to or did not choose to meet ICMJE authorship criteria in on a particular manuscript. Large research teams can specifically offer opportunities to achieve equity and inclusion, given the number of manuscripts to be written and published.

The principles incorporated in the CHARM authorship guidelines reflect the values and challenges identified by team members within our group process. This process led to guidelines that were unique to the CHARM team, yet other large research teams and consortia can engage in a similar process to develop guidelines that serve their needs. The method for creating the guidelines generates opportunities for research teams to collectively engage in a process that can improve collaboration, communication, team dynamics, and the research endeavor.

While these guidelines have been implemented for the CHARM study, there remain several caveats. We did not assess whether these approaches are effective in improving equity, inclusion, and efficiency in manuscript development. We did not review how well the team members adhered to the guidelines. It is unknown whether the use of these guidelines will support resolution of any conflicts between team members. Future research should address the impact of these approaches.

The CHARM team process to develop authorship guidelines used a team science approach to facilitate communication and establish clearer expectations amongst authors and team members in the writing process with a goal to the engagement of the research team. The process used to develop these guidelines and the values identified may be useful to other multi-disciplinary, multi-institutional research teams who face similar challenges.

## Supporting information

Lewis et al. supplementary material 1 Lewis et al. supplementary material

Lewis et al. supplementary material 2 Lewis et al. supplementary material

## Supplementary material

The supplementary material for this article can be found at https://doi.org/10.1017/cts.2023.685.
