# Supplementary material for: Promoting equity, inclusion, and efficiency: A team science approach to the development of authorship guidelines for a multi-disciplinary research team
Source: J Clin Transl Sci. 2023 Nov 30;7(1):e265. doi: 10.1017/cts.2023.685 (PMC10790100; doi:10.1017/cts.2023.685)
Supplement: Lewis et al. supplementary material 1 — Lewis et al. supplementary material [file S2059866123006854sup001.docx]

**Concept Sheet: CHARM Papers**

**Project Title:**Promoting Equity, Inclusion, and Efficiency: A team science approach to the development of authorship guidelines for multi-disciplinary research team

**Lead Author (Affiliation):**

- Hannah Lewis, SCH

**Senior Author(s) (Affiliation):**

- Michael Leo, KPCHR
- Katrina Goddard, NIH
- Ben Wilfond, SCH

**Writing Group Members (Affiliation):**

- Barbara Biesecker, RTI
- Sandra Lee, Columbia

**All Other Authors/ (affiliation):**

- Galen Joseph, UCSF
- Katy Anderson, DH
- Charisma Jenkins, KPNW
- Joanna Bulkley, KPNW

**Target journal:**Journal of Clinical and Translational Science

**Target Date for Submission:**January 2023

**Keywords:**Team Science, Authorship Guidelines, Multi-institutional research, multi-disciplinary research

**Question/Hypothesis:**

Provide direction and guidance for the order, tasks, and responsibilities of authors and co-authors for a manuscript in the context of a multi-institutional study.

**Background/significance:**

Deciding authorship order and responsibilities of a manuscript can pose a difficult task to those involved as there is not a consistent approach to how authorship should be delegated.

The CHARM study has developed a series of recommendations and guidelines to aid in deciding the roles of authors for a manuscript in the context of a multi-institutional, multi-site study. These guidelines can be applied to other studies involving large numbers of investigators, as a process for determining authorship and their execution.

**Summary of project:**

The paper will describe (1) the process by which the recommendation and guidelines were developed (2) the purpose of these guidelines and their utility in defining the roles of senior, lead, and other authors among multiple institutions, and (3) future application of these guidelines and recommendations for other multi-institutional research endeavors.

**Site contributing data:**

All CHARM investigators contributed on some level to the development of these guidelines.

**Concept sheet submission date:**1/15/21
