# Supplementary material for: Promoting equity, inclusion, and efficiency: A team science approach to the development of authorship guidelines for a multi-disciplinary research team
Source: J Clin Transl Sci. 2023 Nov 30;7(1):e265. doi: 10.1017/cts.2023.685 (PMC10790100; doi:10.1017/cts.2023.685)
Supplement: Lewis et al. supplementary material 2 — Lewis et al. supplementary material [file S2059866123006854sup002.pdf]

CHARM Manuscript / Authorship Tool Overview

This document describes the manuscript tracking and authorship distribution tool that was developed for the CHARM study. This tool is mentioned in the “Promoting Equity, Inclusion, and Efficiency: A team science approach to the development of authorship guidelines for a multi-disciplinary research team” manuscript.

The manuscript tracking tool was developed using Smartsheet, a cloud-based work management platform. The template and startup instructions to the tool can be found here: https://app.smartsheet.com/b/launch?lx=IG61eWKGzJYH-i8MNlVaiuqKwon7W423t4KaXJloEug

High Level Features

The CHARM tool was developed with the purpose of tracking manuscripts and authorship distribution more transparently and efficiently.

The template consists of the following elements:

1. Dashboards
    a. Manuscript summary – summarizes progress on manuscripts.
    b. Authorship distribution – graphically shows the distribution of authorship across all papers.
2. Spreadsheets –these sheets contain the source data for dashboards and automations.
    a. Manuscript_MainSheet – Contains the data for each manuscript.
    b. Manuscript: Authorship – Contains the author and affiliation information that is graphically shown on the dashboards.
3. Automations (optional, can choose which are activated)
    a. Update request – Sends a monthly email to first authors of manuscripts with the status of pending or not started, asking them to provide a status update. The email directs the first author to a simple form to fill out to update the information.
    b. Change notifications – Sends an email notification to the designated person when data is changed in the manuscript tracking system.
    c. Congratulations on accepted / published manuscripts – Sends an email to all authors associated with the manuscript for which the status recently changed to “Accepted” and the citation information is entered.
4. Reports – summarizes and organizes data into focused reports that can be exported to Excel or used in dashboards
    a. All Manuscripts – Lists all manuscripts by status
    b. Manuscripts: Accepted/Published – Lists only accepted / published manuscripts
    c. My Manuscripts – Lists only manuscripts where the user is listed as an author (first, senior, or coauthor)
5. Setup guide and data dictionary can also be found in the template (in the “Start Here” dashboard)

Organization

The Smartsheet tracking system developed by the CHARM team is not a relational database. However, the Manuscript: Authorship sheet uses formulas to summarize data from the Manuscript_MainSheet. The sheets are also used to populate the dashboards and reports.

The following schema represents how the data is used in various reports and dashboards.

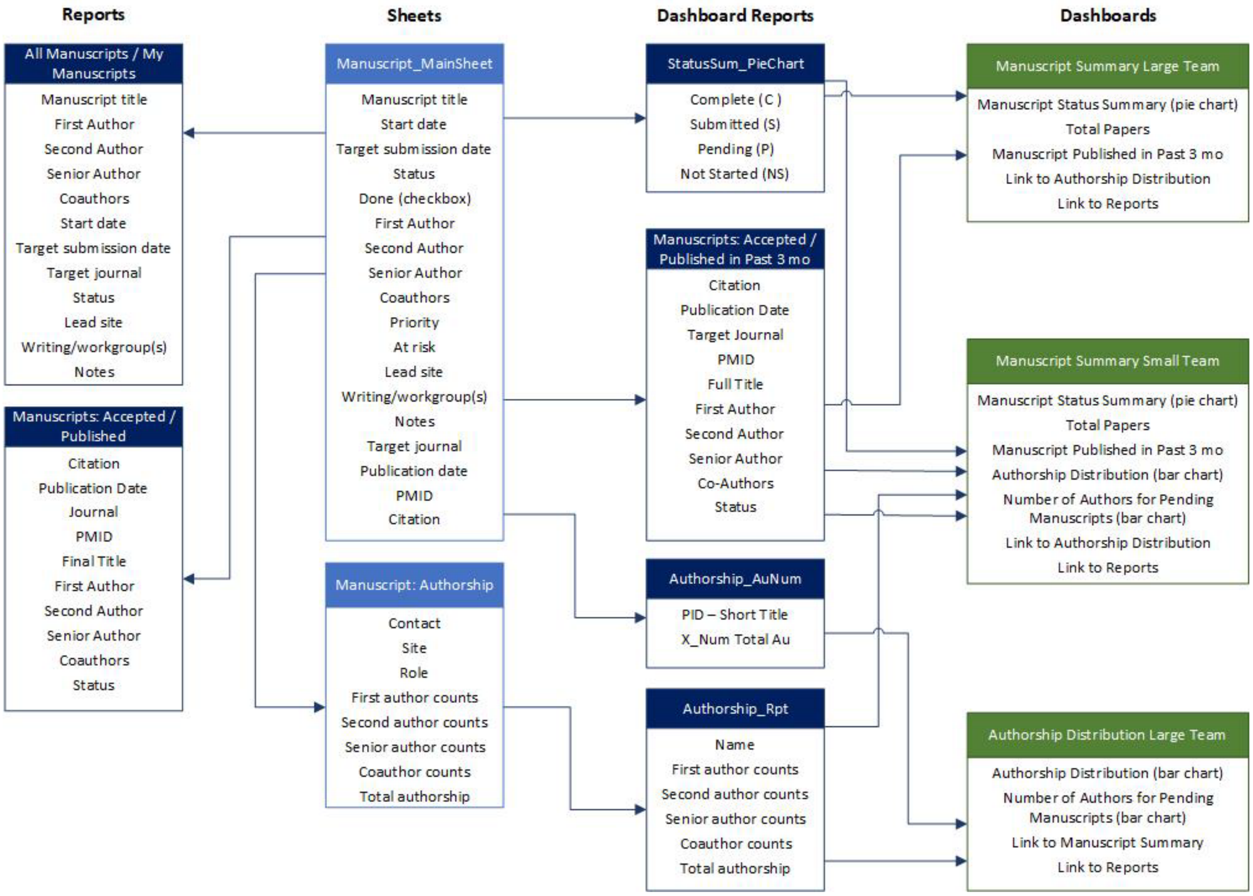

Figure 1 Shows how the sheets connect to other reports and dashboards within the template.

Dashboards

Dashboards provide visual summaries of the information tracked in the data sheets. The CHARM study used these dashboards to share study progress with the research team and to understand authorship distribution.

Depending on the Smartsheet settings, dashboards can be shared with people without Smartsheet accounts or restricted to only people with Smartsheet accounts (free and licensed accounts) with permission to the workspace.

Manuscript Summary – Small Team

This dashboard is meant to summarize the manuscript status information (complete, submitted, pending) and the authorship distribution into one dashboard. If the team is larger, this may not be an ideal dashboard since the authorship distribution graphs list everyone listed as an author.

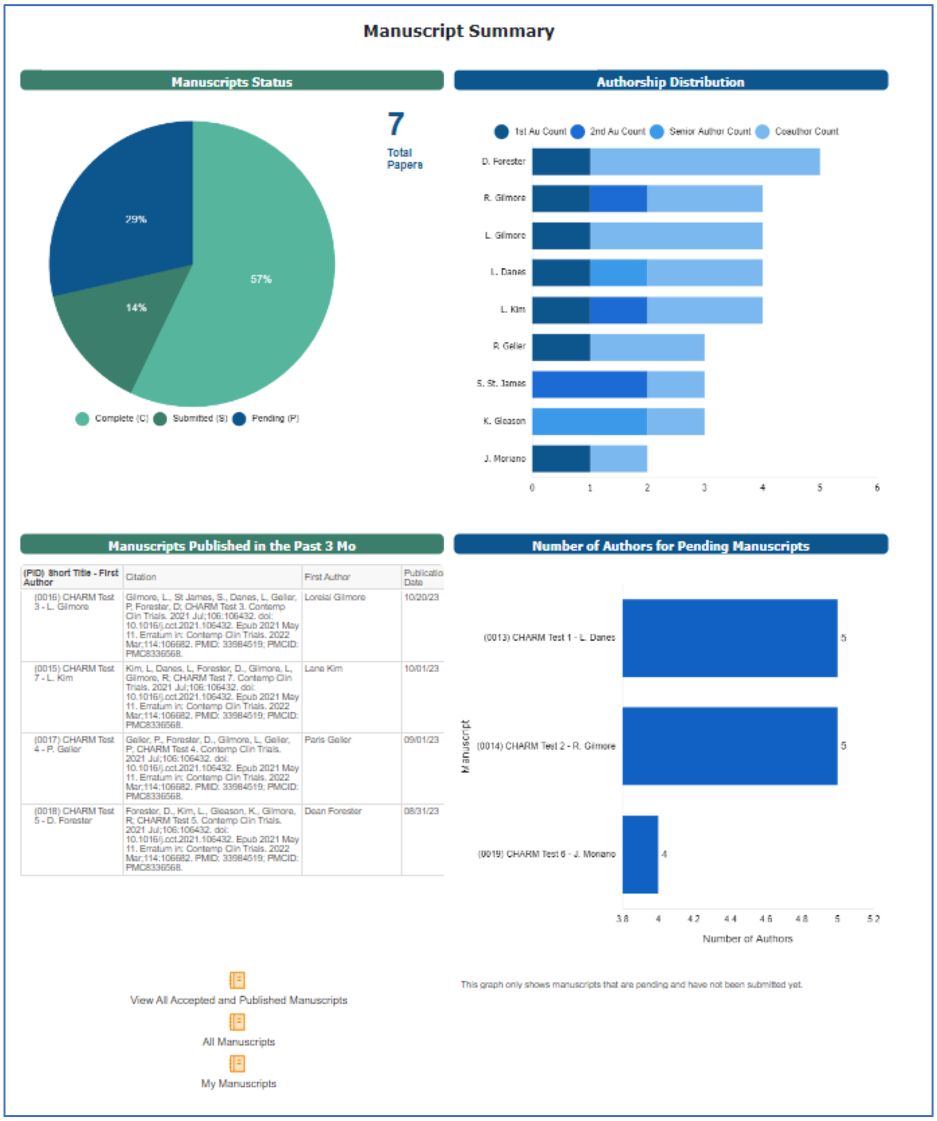

Figure 2 A screenshot of the Manuscript Summary - Small Team dashboard. This screenshot contains fake data.

Manuscript Summary – Large Team

This dashboard is meant to summarize the manuscript status information (complete, submitted, pending). This is a better dashboard for larger teams, since the authorship distribution graph will be larger for larger teams (and therefore better in a separate dashboard – see below).

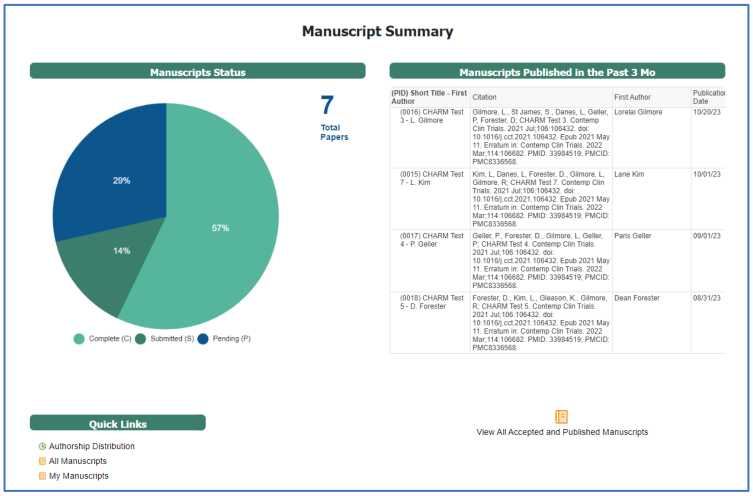

Figure 3 Shows a screenshot of the Manuscript Summary - Large Team dashboard. This screenshot contains fake data.

Authorship Distribution – Large Team

This dashboard shows authorship distribution for the project. This includes a bar graph showing the number and types of authorship opportunities each author on the team is assigned and a bar graph showing the number of authors on each pending manuscript.

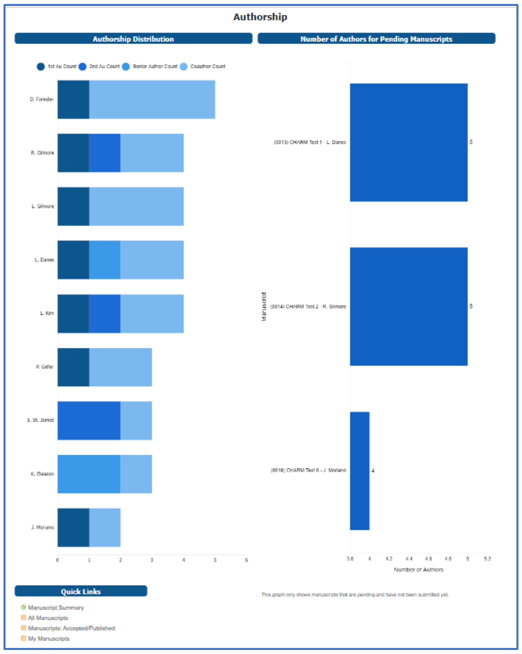

Figure 4 Shows authorship distribution only. This dashboard is better for larger teams. This screenshot contains fake data.

Spreadsheets

Manuscripts_MainSheet

The Manuscripts_MainSheet is the universal truth of manuscript data and all of the manuscript tracking information is saved here. All reports and dashboards are pulling data from this sheet. Manuscripts_MainSheet is also where automations are managed and where the updated information is refreshed.

PID	PID-Title	Short Title	First Author	Full Title	Start Date	Target Submission Date	Status	Done	Status Sum	Status Bar	Impact	Priority/fl...	At Risk	Second Author	Senior Author	Co-Authors
0013	(0013) CHARM Test 1 - L. Danes	CHARM Test 1	Luke Danes	CHARM Test Manuscript: Demoing the Smartsheet Manuscript / Authorship Distribution Tracking Tool	09/01/22	10/16/23	Writing		P					Rory Gilmore		Charisma Jen
0014	(0014) CHARM Test 2 - R. Gilmore	CHARM Test 2	Rory Gilmore	CHARM Test Manuscript: Authorship Distribution	07/15/22	12/01/23	Submitted		S						Kirk Gleason	Lorelai Gilmor
0016	(0016) CHARM Test 3 - L. Gilmore	CHARM Test 3	Lorelai Gilmore	CHARM Test Manuscript 3	07/15/20	08/15/23	Accepted / Published		C					Sookie St. James		Luke Danes
0017	(0017) CHARM Test 4 - P. Geller	CHARM Test 4	Paris Geller	CHARM Test Manuscript 4	08/05/20	12/01/21	Accepted / Published		C						Charisma Jenkins	Dean Foreste
0018	(0018) CHARM Test 5 - D. Forester	CHARM Test 5	Dean Forester	CHARM Test Manuscript 5	09/01/22	04/15/23	Accepted / Published		C					Lane Kim	Kirk Gleason	Rory Gilmore
0019	(0019) CHARM Test 6 - C. Jenkins	CHARM Test 6	Charisma Jenkins	CHARM Test Manuscript 6	07/01/23	01/02/24	Analysis		P					Sookie St. James		Kirk Gleason
0015	(0015) CHARM Test 7 - L. Kim	CHARM Test 7	Lane Kim	CHARM Test Manuscript 7	06/01/20	07/01/21	Accepted / Published		C						Luke Danes	Dean Foreste

Figure 5 shows the first few columns of Manuscripts_MainSheet. This screenshot contains fake data.

Automations

Smartsheet can send automated messages to help with communication and management of data. The templates default to these automations being deactivated, so teams wanting to use this feature will need to follow the setup guide instructions to activate these automations. Messages and fields in the forms can be customized (if the user has the correct permissions and understands how to use Smartsheet).

Depending on the Smartsheet settings, automations can send emails to people without Smartsheet accounts. Users can also restrict automations to only people with Smartsheet accounts (free and licensed accounts). Users should utilize Smartsheet resources available online to understand how to manage permissions and access.

Update Request – Manuscripts (Pending / Not Started) and Update Request – Manuscripts (Pending / Not Started) - Reminder

The automation used most by the CHARM team were update requests on manuscript data. For update requests, the first author is emailed an automated email and asked to update the status of

Hi Charisma Jenkins,

This is our monthly request to all first authors to update information related to pending manuscripts in the Smartsheet tracking system.

We are reaching out to you because you are listed as the first author of the "CHARM Test Manuscript 6" manuscript. Please click on the "Open Update Form" button below to update information related to this manuscript.

Even if you do not have changes to provide, please still open the survey and type "Reviewed (date)" in Notes field before submitting the survey.

Note: Author Lists (First Author, Senior Author, CHARM Co-authors, etc.) = Find the author by typing their name. If you are unable to find their name in the dropdown list, type their full name and press enter (this should be rare, please email us if you need to do this frequently)

If you do not update the record, you will receive a reminder email.

Updating information on this form allows our study to maintain accurate records. This information will be used for reports, including reports to our funding agency. We appreciate your help!

Sincerely,
Study Coordination Team

This is an automated email. If you have questions, please reach out to the SmartSheet administrator

Open request

Figure 6 shows a screenshot of the automated email that can be sent to first authors requesting updates to their manuscript. Once the "open request" button is clicked, a form opens (see figure 7) that allows the first author to update manuscript information without a log-in to Smartsheet.

their manuscript. When the first author clicks the link in the automated email, a form opens with select fields. When the first author enters information and presses submit, the Manuscript_MainSheet data (and associated reports and dashboards) is updated.

The *Update Request – Manuscripts (Pending / Not Started) – Reminder* automation resends the update request email to the first author if the manuscript data is not updated within 7 days.

Change Notifications

The CHARM template also contains an automation that notifies people listed in the “Notify with Changes” cell in the Manuscripts_MainSheet whenever a change is made to the data on the sheet.

Check “Done” When Citation Added and Paper is Published

This automation checks the “done” box automatically when data is entered into the “Citation” and the status = “Accepted / Published” fields in Manuscripts_MainSheet. The “Done” checkbox drives some of the reports and this automated step makes it easier to ensure the field is updated.

Congratulations on Recent Accepted / Published Manuscript

This automated message congratulates the full list of authors on the publication of their manuscript. The body of this email also contains the citation and the PMID.

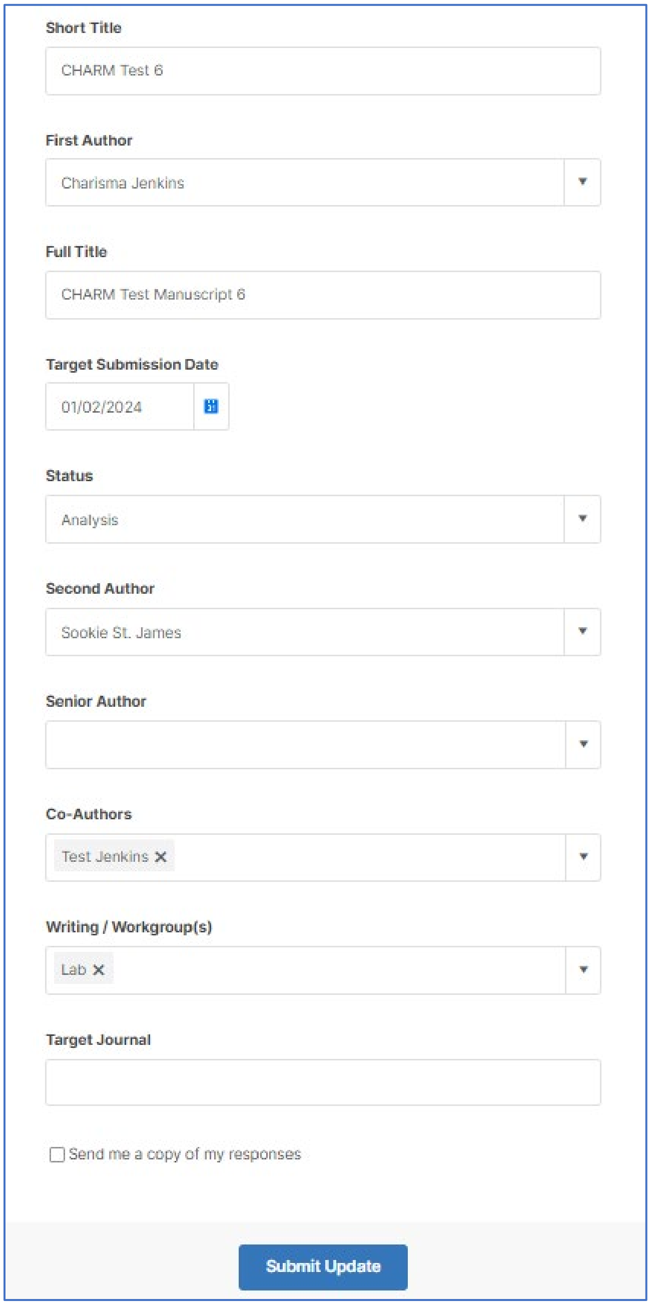

Figure 7 shows the update request form. The first author updates this information. When "submit update" is clicked, the Manuscripts_MainSheet data (and associated reports and dashboards) are updated. This screenshot contains fake data.

Reports

The reports are spreadsheets that organize selected information from the Manuscript_MainSheet into an easier to read report.

All Manuscripts / My Manuscripts

This report shows all manuscripts in the tracking system, grouped by status. The *My Manuscripts* version only shows manuscripts associated with the person that's logged into Smartsheet.

(PID) Short Title - First Author	Short Title	Full Title	First Author	Start Date	Target Submission Date	Target Journal	Status	Status Bar	At Risk	Lead Site	Writing / Workgroup(s)	Notes
**Total**							Count **7**					
Status Sum **C**							Count **4**					
(0017) CHARM Test 4 - P. Geller	CHARM Test 4	CHARM Test Manuscript 4	Paris Geller	08/05/20	12/01/21		Accepted / Published			UW	Lab WG	
(0018) CHARM Test 5 - D. Forester	CHARM Test 5	CHARM Test Manuscript 5	Dean Forester	09/01/22	04/15/23		Accepted / Published			SCH	Patient Engagement	Fake data is here, but here's whe you can add notes
(0015) CHARM Test 7 - L. Kim	CHARM Test 7	CHARM Test Manuscript 7	Lane Kim	06/01/20	07/01/21		Accepted / Published			Baylor	Genetic Counseling	
(0016) CHARM Test 3 - L. Gilmore	CHARM Test 3	CHARM Test Manuscript 3	Lorelai Gilmore	07/15/20	08/15/23		Accepted / Published			KPNW	Risk Assessment	
Status Sum **P**							Count **2**					
(0013) CHARM Test 1 - L. Danes	CHARM Test 1	CHARM Test Manuscript: Demoing the Smartsheet Manuscript / Authorship Distribution Tracking Tool	Luke Danes	09/01/22	10/16/23		Writing			UW	Aim 4	
(0019) CHARM Test 6 - C. Jenkins	CHARM Test 6	CHARM Test Manuscript 6	Charisma Jenkins	07/01/23	01/02/24		Analysis			KPNW	Lab	
Status Sum **S**							Count **1**					
(0014) CHARM Test 2 - R. Gilmore	CHARM Test 2	CHARM Test Manuscript: Authorship Distribution	Rory Gilmore	07/15/22	12/01/23		Submitted			Seattle Childre	Patient Engagement	

Figure 8 shows the first few columns of the All Manuscripts report. This screenshot contains fake data.

Manuscripts: Accepted / Published

Accepted and published papers, sorted by publication date.

(PID) Short Title - First Author	Citation	First Author	Publication Date	Target Journal	PMID	Full Title	Second Author	Senior Author	Co-Authors	Co-Authors Cont	Status	Modified By	Modified
(0016) CHARM Test 3 - L. Gilmore	Gilmore, L., St James, S., Danes, L, Geller, P, Forester, D; CHARM Test 3. Contemp Clin Trials. 2021 Jul;106:106432. doi: 10.1016/j.cct.2021.106432. Epub 2021 May 11. Erratum in: Contemp Clin Trials. 2022 Mar;114:106682. PMID: 33984519; PMCID: PMC8336568.	Lorelai Gilmore	10/20/23	Contemp Clin Trials	33984519	CHARM Test Manuscript 3	Sookie St. James		Luke Danes<br>Paris Geller	Dean Forester	Accepted / Published	charisma.l.jenkins@kp(	10/20/23 3:18 PN
(0015) CHARM Test 7 - L. Kim	Kim, L, Danes, L, Forester, D., Gilmore, L, Gilmore, R; CHARM Test 7. Contemp Clin Trials. 2021 Jul;106:106432. doi: 10.1016/j.cct.2021.106432. Epub 2021 May 11. Erratum in: Contemp Clin Trials. 2022 Mar;114:106682. PMID: 33984519; PMCID: PMC8336568.	Lane Kim	10/01/23	JCTS	33984519	CHARM Test Manuscript 7		Luke Danes	Dean Forester<br>Lorelai Gilmore	Rory Gilmore	Accepted / Published	charisma.l.jenkins@kp(	10/20/23 3:17 PN
(0017) CHARM Test 4 - P. Geller	Geller, P., Forester, D., Gilmore, L, Geller, P; CHARM Test 4. Contemp Clin Trials. 2021 Jul;106:106432. doi: 10.1016/j.cct.2021.106432. Epub 2021 May 11. Erratum in: Contemp Clin Trials. 2022 Mar;114:106682. PMID: 33984519; PMCID: PMC8336568.	Paris Geller	09/01/23	PHG	33984519	CHARM Test Manuscript 4		Charisma Jenkins	Dean Forester<br>Lorelai Gilmore	Paris Geller	Accepted / Published	charisma.l.jenkins@kp(	10/20/23 3:17 PN
(0018) CHARM Test 5 - D. Forester	Forester, D., Kim, L., Gleason, K., Gilmore, R; CHARM Test 5. Contemp Clin Trials. 2021 Jul;106:106432. doi: 10.1016/j.cct.2021.106432. Epub 2021 May 11. Erratum in: Contemp Clin Trials. 2022 Mar;114:106682. PMID: 33984519; PMCID: PMC8336568.	Dean Forester	08/31/23	JCTS	33984519	CHARM Test Manuscript 5	Lane Kim	Kirk Gleason	Rory Gilmore		Accepted / Published	charisma.l.jenkins@kp(	10/20/23 3:17 PN

Figure 9 show the Manuscripts: Accepted / Published report. This screenshot contains fake data.

Smartsheet Manuscript Tracking

Using the CHR manuscript tracking template assets, this version was created by the CHARM study.

Created: February 22, 2022
Created by: Charisma Jenkins
(Kaiser Permanente - Center for Health Research, Department of Translational and Applied Genomics)

Version Number: 1.0
Last Revised: November 8, 2022

Table of Contents

Purpose 2
Using this Document 2
SmartSheet Template 2
Save a New Copy of the SmartSheet Template 2
Overview of Template 2
Manuscript – Main Sheet 3
Orientation of Main Sheet 3
Setup and Customization of Manuscript – Main Sheet 3
Importing Existing Data to the Main Sheet 4
Viewing Data 4
Automations 4
Activating Automation Workflows 5
Modifying Automation Workflows 5
Authorship Distribution 5

Purpose

The purpose of this document is to outline some ideas on manuscript tracking template (complex version) can be used and step-by-step instructions on how to modify the Smartsheet template for their own purposes.

Using this Document

Sections with the icon indicates a step required to setup and customize the SmartSheet for your study.

Smartsheet Template

Save a New Copy of the Smartsheet Template

Before using the Smartsheet template for another study, save a copy on your own Workspace.

1. Right click on "Manuscript Tracking (CHARM Template) and select "Save as New"
2. Rename folder to desired name and select "Browse" button to save to desired Smartsheet location

Overview of Template

The entire manuscript tracking template includes multiple Smartsheet assets to enable features in its entirety. Here is a list of Smartsheet Assets:

**Name**	**Description**	**Location**
Manuscript_MainSheet	This is the sheet that drives most of the dashboards and reports and where most of the tracking occurs. This sheet also controls the automated messages.	Complex Tracking Folder
Manuscript: Authorship	This sheet cross references Manuscript_MainSheet and tallies the number of authorship opportunities listed for each author (note: you will need to enter the names of all the authors on your team in the "Contact" column – see instructions below under Authorship Distribution).	Other Source Files
Submitted or Accepted – No Citation	A list of manuscripts that have either been submitted or accepted, but citation has not been entered.	Reports
My Manuscripts	Shows a list of manuscripts that have the current users as either first, second, senior, or coauthor.	Reports
All Manuscripts	Shows a list of manuscripts, grouped by status. If the user clicks on the pie chart, this is the report that appears.	Reports
Manuscript_AuthorshipRpt	Uses the Manuscript: Authorship sheet to create a summary of the number of authorship opportunities per person. This is the report that is referenced for the dashboard graphs.	Reports\ Dashboard Reports
Manuscripts: Accepted/Published	A list of all Accepted/Published manuscripts, which is linked to from the dashboard for quick reference.	Reports\ Dashboard Reports

Manuscripts: Accepted/Published in Past 3 mo.	Shows the list of manuscripts published in the past 3 mo.	Reports\ Dashboard Reports
StatusSum_PieChart	This report is referenced for the pie chart on the manuscript dashboard.	Reports\ Dashboard Reports
Manuscript Summary – Small Team	High level dashboard that shows the executive level summary of manuscript status and authorship distribution.<br><br>Small team = authorship distribution is on this dashboard instead of separated to another dashboard	Dashboard
Manuscript Summary – Large Team	High level dashboard that shows the executive level summary of manuscript status.<br><br>Large team = authorship distribution is on a separate dashboard instead on the summary dashboard to accommodate a larger authorship graph.	Dashboard
Authorship Distribution – Large Team	High level dashboard that shows authorship distribution. This dashboard has a link to the "Manuscript Summary – Large Team" dashboard and is meant to be used with that dashboard.	Dashboard
Data Dictionary	Data dictionary for the system, includes explanations of variables and automations on sheets.	Template Resources

Manuscript – Main Sheet

This will be the primary sheet tracking the manuscripts on the project. This sheet will drive most of the reports, dashboards, and automated actions.

Orientation of Main Sheet

The main sheet should contain everything that you want to track related to manuscripts. For the sake of simplicity, we have designed each row to be its own manuscript without child rows.

Go to Data Dictionary in Smartsheet template folder for more information on the fields in the Smartsheet (Smartsheet Vanguard Group -> Manuscript Tracking -> Other Source Files).

In addition, click on the icon for more information about that column.

Setup and Customization of Manuscript – Main Sheet

1. Contact Lists (First Author, Second Author, Senior Author, Co-Authors, Co-Authors Cont, Notify with Changes) – *optional, but recommended if you are requesting many people on your team to update this information either directly on the sheet or via automated update requests*
    a. Bulk upload of contacts – follow steps on Smartsheet Help & Learning's Manage contact in the My Smartsheet Contacts list
    b. Modify the column properties to create a Contact List dropdown list

        i. Right-click the column header -> Edit Column Properties…
        ii. Values (Optional) – enter the names you want listed on the dropdown list
        iii. Check the “Restrict to list values only” if desired
        iv. Press OK
    c. Save Sheet
2. Lead Site dropdown (single select) – *optional, user can type in any value if not customized and values restricted*
    a. Right-click the column header -> Edit Column Properties…
    b. Enter site names (as you want them to appear on the tracking sheet) under Values
    c. Check the “Restrict to list values only” if desired
    d. Press OK
    e. Save Sheet
3. Writing/Workgroup(s) (multi-select) - *optional, user can type in any value if not customized and values restricted* – see Lead Site dropdown directions
4. Conditional Formatting – *optional* – See module on [Smartsheet Learning Center]
5. Automations – see below
6. Sorting and Filtering – *optional* (best to do after you have data entered) – See module on the [SmartSheet Learning Center]
    a. Filters can be helpful if you are looking at data in a different view. Filters can be shared with everyone with access to the Smartsheet.

Importing Existing Data to the Main Sheet

If applicable, import existing data to the manuscript sheet using the following steps. If you want to customize this sheet by adding more columns, add the columns before taking these steps.

1. Structure data on your source document (Excel spreadsheet) the same order as the Smartsheet Main Sheet
    a. (optional) Export the Smartsheet to Excel to help create the source document by going to File -> Export -> Export to Microsoft Excel
    b. If there is a column on the sheet that you have no data, leave this cell blank
    c. Do not worry about having data for the following columns (Smartsheet will create these for you): PID, PID-Title, Done, Status Sum, Status Bar, At Risk, Short Title (PID), Modified By, Modified, Hierarchy, X_Num columns, X_Last Name, Published Quarter
2. Starting with “Short Title” column (skipping the PID and PID-Title columns), copy all rows and paste into the Manuscript_MainSheet
3. Review all changes (use ⟲ if anything pasted into the wrong column)
4. Save Smartsheet
5. Type all Contact List columns (First Author, Second Author, Senior Author, Co-Authors, Co-Authors Cont, Notify with Changes) to update columns
    a. This step is especially important if you are planning to examine authorship distribution

Viewing Data

View data in [Card View], [Basic Gantt View], and [Calendar View] if desired.

Automations

The Manuscript_MainSheet has automated messages that can activated (the default will be that all automations will be unactive) and modified to the needs of the project.

Activating Automation Workflows

Automations (such as automated messages asking first authors for updates to their manuscripts) will not happen until the automation workflows are activated. Activate workflow by:

1. Manuscript_MainSheet -> Automation (in menu) -> Manage Workflows
2. Next to the workflow of choice, select ⋮ and "Activate" toggle

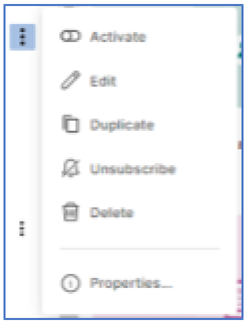

*Figure SEQ Figure * ARABIC 1: Activate the automation workflow by selecting the "Activate" toggle*

Studies may not choose to activate all workflows. See the section below for an explanation of the workflows that come with the template to have information that can help your team decide which automations work best for your study.

Modifying Automation Workflows

You may want to customize the messages, change the triggers of automation, or change the recipient of messages. If you decide to use automation, it is recommended to review all automations and modify them as desired.

Authorship Distribution

If the study hopes to use the authorship distribution graphs on the dashboards, the Manuscript: Authorship sheet will need to updated.

Setup and Customization of Manuscript: Authorship

Modify applicable column properties:

1. Contact
    a. See instructions on how to modify the column properties under the **Setup and Customization of Manuscript – Main Sheet**
2. Site – *optional, user can type in any value if not customized and values restricted*
    a. Right-click the column header -> Edit Column Properties…
    b. Enter site names (as you want them to appear on the tracking sheet) under Values
    c. Check the "Restrict to list values only" if desired
    d. Press OK
    e. Save Sheet
3. Role – *optional, user can type in any value if not customized and values restricted* – use the same process as "site"
    a. Value options default to "Site PI," "Current," and "Former"

Complete data entry: Add all study teammates to the Contact column. In addition, enter optional fields as desired.

- Note: there is no way to enter contacts on mass, unlike other field types. Manual entry during setup is the only method of entering these data.

Manuscript Tracking Data Dictionary

Variable	Description	Variable Type
Manuscript_MainSheet Variables		
PID	Tracking number used to help track papers. This number is generated automatically by Smartsheet.	Auto-Number/System
PID-Title	Combines the short title, PID, and first author name into one field for ease of referencing on reports.	Text/Number
Short Title	Short Title of the manuscript that's easier for referencing. For tasks (child rows of the manuscript - optional on this sheet) this will be the name of the task.	Text/Number
First Author	In the context of the tracking system, we only allow 1 first author (even if there is more than one first author). This person will be asked to provide updates to the tracking system monthly (if automation feature	Contact List
Full Title	Full title of the manuscript. Early in the manuscript development process, it's ok for this to be a working title. After the manuscript is submitted, this should match the title sent to the journal.	Text/Number
Start Date	Start date of when the manuscript is tracked (this can be when the manuscript topic is first identified, when the concept sheet is submitted, etc.).	Date
Target Submission Date	The planned date of submission, this will not necessarily be the actual submission date. This is also different from the Publication Date (another field of this sheet). This date should be used for management and coordination of manuscripts.	Date
Status	Status of the manuscript: potential, concept, planning/design, analysis, writing, submission prep, submitted, rejected/resubmit queue, accepted/published, canceled/combined	Dropdown (Single Select)
Done	This box should be checked if the manuscript has been Accepted/Published and all other information added to tracking. This box can be checked via an automation (if the study activates that feature).	Checkbox
Status Sum	This field summarizes the "Status" field into a high-level classification. The categories are NS (Not Started), P (Pending), S (Submitted), C (Complete), X (Canceled).	Text/Number
Status Bar	Visual representation of the stage of the manuscript.	Symbols...
Impact	Can be used to prioritize manuscripts and tasks. High, medium and low impacts can be designated.	Symbols...
Priority/Flag	Can be used by workgroups or teams to assign numeric priority designations to help manage workload.	Text/Number
At Risk	If the "End Date" (aka the due date for tasks or the target submission date for manuscripts) is in the past AND the "Done" checkbox is unchecked, this flag will be	Symbols...
Second Author	Second author of the manuscript. Only one author is allowed in this field.	Contact List
Senior Author	Senior Author(s), multiple authors are allowed in this field. They are listed in alphabetical order by first name.	Contact List
Co-Authors	Co-Author(s), multiple people allowed. Listed in alphabetical order by first name	Contact List
Co-Authors Cont	Co-Author(s), multiple people allowed. Listed in alphabetical order by first name. Use this column if the "Co-authors" column has over 20 authors listed (the max allowed in contact lists)	Contact List

Manuscript Tracking Data Dictionary

Variable	Description	Variable Type
Notify with Changes	Person/people that should be notified if there are any changes to the associated row (if automation created and/or activated)	Contact List
Lead Site	The site of the first author (for studies with multiple sites)	Dropdown (Single Select)
Primary Analysis	Qualitative and quantitative primary analysis types	Dropdown (Single Select)
Writing/Workgroup(s)	Writing or Working group that is sponsoring this manuscript	Dropdown (Multi Select)
Notes	Notes that are entered into the tracking system	Text/Number
Target Journal	Journal that the first author plans on submitting the paper or the Journal that the manuscript was published.	Text/Number
Publication Date	Date manuscript was published.	Date
PMID	PMID of published manuscript	Text/Number
Citation	Citation of published manuscript	Text/Number
Short Title (PID)	Combination of short title and the PID, this is used for reports and dashboards	Text/Number
Modified By	Automated field that tracks the last person to make changes to this row.	Text/Number
Modified	Automated field that shows the time the last change was made to this field.	Text/Number
Hierarchy	This field is used to power a lot of reports and is useful if a study tracks tasks as child rows. Hierarchy = 0 is a parent row, or the row containing the paper level information. Hierarchy = 1 is a child row, which are the rows that track manuscript tasks	Text/Number
X_Num First Au	A count of first authors. Used for reporting	Text/Number
X_Num Second Au	A count of second authors. Used for reporting	Text/Number
X_Num Senior Au	A count of Senior Authors. Used for reporting.	Text/Number
X_Num Coauthors	A count of Coauthors (includes co-authors from "Co-authors" column and "Co-authors cont")	Text/Number
X_Num Total Au	A count of total authors listed on the manuscript, used for one of the dashboard reports.	Text/Number
X_Last Name	Extracts the first authors last name and first initial. Used for the PID-Title field.	Text/Number
PublishedQuarter	Quarter that the manuscript was published. In the template, not used for any dashboards or reports, but we kept this field because it might be useful for future reports that the SmartSheet admin wants to create.	Text/Number
Automation Workflow - Manuscript_MainSheet		
Change Notification	When the status, target submission date, first author, or target journal is changed, a message is sent to people listed in the “Notify with Changes” cell.	
Check Done when Citation Added and Paper Published	After text is entered in the “Citation” field for manuscripts with a status of “Accepted/Published”, the “Done” checkbox is automatically checked. This drives some of the reports that help manage which manuscripts have the data entry completed related to published	
Congratulations on Recent Accepted/Published	When the status is “Accepted/Published” and “Done” is checked, a congratulations message is sent to everyone listed as authors and provides everyone with the citation entered in the SmartSheet.	

Manuscript Tracking Data Dictionary

Variable	Description	Variable Type
Update Request – Manuscripts (Pending/Not Started)	Sends a monthly email to the first author requesting updates to the manuscript. This includes the following fields: full title, target submission date, authorship fields, status, target journal, writing/workgroup, short title	
Update Request – Manuscripts (Pending/Not	Sends reminder email requesting updates to first authors that did not respond to the first request	
Manuscript: Authorship		
Last Name	Extracts the last name from the Contact. This will be used for some of the graphs.	Text/Number
Contact	Name of the study teammate. This field is used to reference the Manuscript_MainSheet when calculating the counts.	Contact List
Site	Optional, site of the study teammate	Dropdown (Single Select)
Role	Optional, track if person is a site PI, former, or current study teammate	Dropdown (Single Select)
1st Au Count	For the person listed in the "Contact" field, how many times are they listed as first author on a manuscript. This counts all manuscripts except ones with a status of "X" (Canceled/Combined)	Text/Number
2nd Au Count	For the person listed in the "Contact" field, how many times are they listed as second author on a manuscript. This counts all manuscripts except ones with a status of "X" (Canceled/Combined)	Text/Number
Senior Au Count	For the person listed in the "Contact" field, how many times are they listed as senior author on a manuscript. This counts all manuscripts except ones with a status of "X" (Canceled/Combined)	Text/Number
Coauthor Au Count	For the person listed in the "Contact" field, how many times are they listed as coauthor on a manuscript. This counts all manuscripts except ones with a status of "X" (Canceled/Combined)	Text/Number
Total Authorship	For the person listed in the "Contact" field, how many times are they listed as any kind of author on a manuscript. This counts all manuscripts except ones with a status of "X" (Canceled/Combined)	Text/Number
Coauthor Column	For the person listed in the "Contact" field, how many times are they listed in the coauthor column on a manuscript. This is combined with the Coauthor Cont Column to count total coauthor lists. This counts all manuscripts except ones with a status of "X" (Canceled/Combined)	Text/Number
Coauthor Cont Column	For the person listed in the "Contact" field, how many times are they listed in the coauthor column on a manuscript. This is combined with the Coauthor Column to count total coauthor lists. This counts all manuscripts except ones with a status of "X" (Canceled/Combined)	Text/Number
